# Supplementary material for: Anharmonic quantum nuclear densities from full dimensional vibrational eigenfunctions with application to protonated glycine
Source: Nat Commun. 2020 Aug 28;11:4348. doi: 10.1038/s41467-020-18211-3 (PMC7455743; doi:10.1038/s41467-020-18211-3)
Supplement: Supplementary file 2 — Supplementary Information [file 41467_2020_18211_MOESM2_ESM.pdf]

Anharmonic quantum nuclear densities from full dimensional vibrational eigenfunctions with application to protonated glycine

Aieta *et al.*

SUPPLEMENTARY METHODS

The vibrational frequencies presented in the main text have been determined upon calculation of power spectra of the type

$$\tilde{I}_\chi(E) = \frac{1}{\pi\hbar} Re \int_0^\tau dt \, \langle\chi| \, e^{-\frac{i}{\hbar}\hat{H}t} \, |\chi\rangle \, e^{\frac{i}{\hbar}Et} \tag{1}$$

in semiclassical approximation. In Supplementary Equation 1 the quantum state $|\chi\rangle$ is evolved by the quantum nuclear dynamics operator $e^{-\frac{i}{\hbar}\hat{H}t}$ at time t and overlapped with itself at time zero. The Fourier transform of this autocorrelation function is the power spectrum and it reproduces all vibrational energy levels, including the zero point energy (ZPE) one. The semiclassical propagator of the time-evolution operator $e^{-\frac{i}{\hbar}\hat{H}t}$ for the quantum Hamiltonian $\hat{H}$ is obtained by approximating the exact quantum propagator expressed in terms of Feynman's path integral. In Feynman's path integral formulation[1] the quantum mechanical probability amplitude of going from an initial state $\mathbf{Q}_0$ to a final one $\mathbf{Q}_t$ at time t is obtained by summing up over all the paths that connect the two states

$$\left\langle \mathbf{Q}_t \left| e^{-i\hat{H}t/\hbar} \right| \mathbf{Q}_0 \right\rangle = \int_{\mathbf{Q}_0}^{\mathbf{Q}_t} \mathcal{D}\left[\mathbf{Q}(t)\right] e^{iS_t[\mathbf{Q}_0,\mathbf{Q}_t]/\hbar} \tag{2}$$

where a complex weight that depends on the path action S_t is associated to each path and $\mathcal{D}\left[\mathbf{Q}(t)\right]$ is the differential of the sum over all possible paths. Upon stationary-phase approximation, obtained by expanding the action up to the second order around each path $\mathbf{Q}(t)$ and enforcing $\delta S_t/\delta\mathbf{Q}(t) = 0$, the integral in Supplementary Equation 2 becomes a sum over all possible classical trajectories starting from $\mathbf{Q}_0$ and ending at $\mathbf{Q}_t$ at time t. The result of this approximation is the van Vleck version of the semiclassical propagator,[2] which is reported in Supplementary Equation 3.

$$\left\langle \mathbf{Q}_t \left| e^{-i\hat{H}t/\hbar} \right| \mathbf{Q}_0 \right\rangle \approx \sum_{\text{cl. paths}} \sqrt{\frac{1}{(2\pi i\hbar)^{N_v}} \left| -\frac{\partial^2 S_t^{cl}(\mathbf{Q}_0,\mathbf{Q}_t)}{\partial\mathbf{Q}_t \partial\mathbf{Q}_0} \right|} e^{iS_t^{cl}(\mathbf{Q}_0,\mathbf{Q}_t)/\hbar - i\nu\pi/2}$$

$$= \sum_{\text{cl. paths}} \sqrt{\frac{1}{(2\pi i\hbar)^{N_v}} \left| \frac{\partial\mathbf{Q}_t}{\partial\mathbf{p}_0} \right|^{-1}} e^{iS_t^{cl}(\mathbf{Q}_0,\mathbf{Q}_t)/\hbar - i\nu\pi/2}, \tag{3}$$

where N_v is the number of the vibrational degrees of freedom, $\mathbf{p}_0$ is the initial momentum of the classical path, and v is the Maslov index, which ensures the continuity of the complex square root. The double-boundary values problem of Supplementary Equation 3, which is given by finding the classical trajectory starting at $\mathbf{Q}_0$ and ending at $\mathbf{Q}_t$ at time t, is quite cumbersome. It is with the introduction of the Initial Value Representation (IVR)[3] by W. H. Miller that the semiclassical approximation (SCIVR) becomes computationally feasible. Application of the SCIVR propagator to the survival amplitude of a generic reference wavefunction $|\chi\rangle$ leads to

$$\left\langle \chi \left| e^{-i\hat{H}t/\hbar} \right| \chi \right\rangle \approx \int\int d\mathbf{p}_0 d\mathbf{Q}_0 \sqrt{\frac{1}{(2\pi i\hbar)^{N_v}} \left| \frac{\partial\mathbf{Q}_t}{\partial\mathbf{p}_0} \right|} \chi^*(\mathbf{Q}_t)\, \chi(\mathbf{Q}_0)\, e^{iS_t^{cl}(\mathbf{p}_0,\mathbf{Q}_0)/\hbar - i\nu\pi/2}. \tag{4}$$

Supplementary Equation 4 has two main advantages. One is to substitute the double-boundary problem by the easy generation of classical trajectories starting from their initial phase-space conditions $(\mathbf{p}_0, \mathbf{Q}_0)$. And the other is the removal of the partial derivative at the denominator of Supplementary Equation 3 which leads to an unphysical divergence of the propagator at the cusp (i.e. multidimensional turning) points.

The introduction of the coherent states representation by E. Heller[4, 5] makes the SCIVR formulation even more computationally amenable. Coherent states are suitable to describe both bound and unbound systems since their projection onto the coordinate space consists in a Gaussian-shaped real part and a free-particle imaginary part as follows

$$\langle \mathbf{x} \,|\mathbf{p}_t\mathbf{Q}_t\rangle = \left(\frac{\det(\Gamma)}{\pi^F}\right)^{\frac{1}{4}} e^{-\frac{1}{2}(\mathbf{x}-\mathbf{Q}_t)^T\Gamma(\mathbf{x}-\mathbf{Q}_t) + \frac{i}{\hbar}\mathbf{p}_t^T(\mathbf{x}-\mathbf{Q}_t)}. \tag{5}$$

In our vibrational calculations, the width of the multidimensional coherent state in normal mode coordinates is chosen to be a diagonal matrix (Γ) and its diagonal elements are equal to the square roots of the eigenvalues of the mass-scaled Hessian matrix at the equilibrium geometry, *i.e.* the harmonic frequencies. The resulting semiclassical propagator in the coherent state representation is the Herman Kluk (HK) propagator[6] and it is equal to

$$\left\langle \chi \left| e^{-i\hat{H}t/\hbar} \right| \chi \right\rangle \approx \left(\frac{1}{2\pi\hbar}\right)^F \iint d\mathbf{p}_0 d\mathbf{Q}_0 C_t(\mathbf{p}_0,\mathbf{Q}_0)\, e^{\frac{i}{\hbar}S_t(\mathbf{p}_0,\mathbf{Q}_0)} \langle\chi\,|\mathbf{p}_t\mathbf{Q}_t\rangle\langle\,\mathbf{p}_0\mathbf{Q}_0|\,\chi\rangle, \tag{6}$$

where the cl apex has been dropped from the classical action S_t and the pre-exponential factor - $C_t(\mathbf{p}_0, \mathbf{Q}_0)$ - is the second order derivative of the action in Supplementary Equation 3 in coherent state representation. This quantity is quite important since it accounts for quantum effects. However, it is also difficult to calculate because it is affected by the possible chaotic behavior of the classical trajectories initiated from the phase space points $(\mathbf{p}_0, \mathbf{Q}_0)$, i.e it is sensitive to the initial phase space conditions. Its expression is

$$C_t(\mathbf{p}_0, \mathbf{Q}_0)=\sqrt{\det\left[\frac{1}{2}\left(\mathbf{M_{QQ}} + \mathbf{\Gamma}^{-1}\mathbf{M_{pp}}\mathbf{\Gamma} + \frac{i}{\hbar\mathbf{\Gamma}}\mathbf{M_{pQ}} - i\hbar\mathbf{\Gamma}\mathbf{M_{Qp}}\right)\right]}, \tag{7}$$

where $\mathbf{M_{ij}} = \partial\mathbf{i}_t/\partial\mathbf{j}_0 \quad (\mathbf{i},\mathbf{j} = \mathbf{p},\mathbf{Q})$ is itself a matrix which represents a generic element of the $2N_v \times 2N_v$ dimensional monodromy (or stability) matrix[7, 8]. Specific algorithms exist to reduce the computational effort for the prefactor calculation induced by the presence of the Hessian for the classical time-evolution of the monodromy matrix elements.[9–11]

Eventually, the SCIVR approximation to the power spectrum of Supplementary Equation 1 is

$$\tilde{I}_\chi(\mathrm{E})=\left(\frac{1}{2\pi\hbar}\right)^{N_v+1}\int_{-\infty}^{+\infty}\mathrm{d}t\, e^{iEt/\hbar}\iint \mathrm{d}\mathbf{p}_0\mathrm{d}\mathbf{Q}_0 C_t(\mathbf{p}_0, \mathbf{Q}_0)\, e^{\frac{i}{\hbar}S_t(\mathbf{p}_0,\mathbf{Q}_0)}\,\langle\chi\,|\mathbf{p}_t\mathbf{Q}_t\,\rangle\langle\,\mathbf{p}_0\mathbf{Q}_0|\,\chi\rangle. \tag{8}$$

Unfortunately, this formulation is difficult to converge and it is computationally very demanding.[12, 13] To overcome this issue, Kaledin and Miller demonstrated that it is possible to introduce a time-averaging (TA) filter in Supplementary Equation 8 that reduces the computational effort in phase space. The final expression for the spectral density in Supplementary Equation 8 becomes

$$\tilde{I}_\chi(\mathrm{E})=\left(\frac{1}{2\pi\hbar}\right)^{F}\iint \mathrm{d}\mathbf{p}_0\mathrm{d}\mathbf{Q}_0\frac{1}{2\pi\hbar T}\left|\int_0^T dt e^{\frac{i}{\hbar}[S_t(\mathbf{p}_0,\mathbf{Q}_0)+Et+\phi(t)]}\,\langle\chi\,|\mathbf{p}_t\boldsymbol{Q}_t\,\rangle\right|^2. \tag{9}$$

where the phase-space integrand is positive-definite and, consequently, the integral is much easier to converge and ϕ_t is the phase of the Herman-Kluk prefactor.

However, Supplementary Equation 9 still needs about a thousand of classical trajectories per degree of freedom to reach numerical convergence. This amount is not feasible for direct ab initio dynamcs, i.e. “on-the-fly”, approaches, which are necessary for large molecular systems. Specifically, on-the-fly simulations must rely on a limited number of trajectories to be computationally affordable. To this end, we recently introduced a technique known as Multiple Coherent Semiclassical Initial Value Representation (MC SCIVR), where one trajectory is tailored for each reference state $|\chi\rangle$. The trajectories are run at an energy corresponding to the harmonic estimate for the generic n-th state to investigate, while the corresponding reference state is chosen as $\left|\chi^{(n)}\right\rangle = |\phi_{\boldsymbol{K}}\rangle$. In normal mode representation, the latter reads as

$$\langle\boldsymbol{Q}|\phi_{\boldsymbol{K}}\rangle = G(\mathbf{Q},\mathbf{\Gamma})\bar{\phi}_{\mathbf{K}}(\mathbf{Q}) \tag{10}$$

where $G(\mathbf{Q},\mathbf{\Gamma}) = |\mathbf{\Gamma}/(\pi\hbar)|^{1/4}\exp\left(-\mathbf{Q}^T\mathbf{\Gamma}\mathbf{Q}/(2\hbar)\right)$, and $\bar{\phi}_{\mathbf{K}}(\mathbf{Q}) = \prod_{\alpha=1}^{N_v}\left(2^{K_\alpha}K_\alpha!\right)^{-1/2}\ h_{K_\alpha}\left(\sqrt{\omega_\alpha/\hbar}\,Q_\alpha\right)$, with the $\mathrm{K}^{th}{}_\alpha$-order Hermite polynomial h_{K_α}. In these expressions, $\mathbf{\Gamma}$ is a 3N $\times$ 3N diagonal matrix whose diagonal elements are the eigenvalues ω_α ($\alpha = 1,\ldots,3N$) obtained from the diagonalization of the mass-scaled Hessian at equilibrium. The harmonic states in Supplementary Equation 10 are centered at the equilibrium geometry and their quantum excitation numbers $\boldsymbol{K}$ determine the initial conditions and the energy of the classical trajectories needed by the classical simulation. Their parity enforces molecular symmetries, and allows for highlighting specific fundamental excitations or overtones. The main advantage brought in by MC SCIVR is that accurate results can be obtained by running just one tailored classical trajectory $\left(\mathbf{p}_0^{(n)},\mathbf{Q}_0^{(n)}\right)$ per state with the effect to alleviate the computational cost substantially.[14–17] The trajectory initial conditions corresponding the n-th vibrational state is tailored via the Einstein-Brillouin-Keller (EBK) rules

$$\oint_{H\left(\mathbf{Q}_0^{(n)},\mathbf{p}_0^{(n)}\right)=E_n}\boldsymbol{p}^{(n)}d\boldsymbol{Q}^{(n)} = \hbar\left(\zeta_n + \frac{\mu_n}{4}\right) \tag{11}$$

where ζ_n are positive integers, and μ_n are Maslov indexes.[18] In the separable case, these rules provide a link between the n-th vibrational state and a N_v-dimensional vector of natural numbers $\boldsymbol{\nu}$, such that $\zeta_n = \sum_\alpha \nu_\alpha$, which is valid also beyond the harmonic approximation. In the MC-SCIVR approach, these classical trajectories are chosen with total energy (and energy partition) corresponding to the harmonic oscillator spectral energies $E_{\boldsymbol{\nu}}^{HO} = \sum_\alpha(1/2+\nu_\alpha)\hbar\omega_\alpha$, and are generated by considering the initial conditions

$$Q_{0,\alpha}^{(n)} = \sqrt{\frac{2\nu_\alpha + 1}{\hbar\omega_\alpha}}\sin(\delta_\alpha)$$
$$p_{0,\alpha}^{(n)} = \sqrt{(2\nu_\alpha + 1)\hbar\omega_\alpha}\cos(\delta_\alpha), \tag{12}$$

where the angles δ_α govern the partition of the starting energy of the α-th normal mode into potential and kinetic terms.

By calling $\boldsymbol{R}_i$ the Cartesian coordinates of the i-th nucleus in the molecule, the associated marginal one-nucleus density is

$$\rho_{n,\boldsymbol{R}_i}(\boldsymbol{R}) = \int d^{3N}\boldsymbol{Q}\, |\langle \boldsymbol{Q}|e_n\rangle|^2\, \delta(\boldsymbol{Q}^{RT})\delta(\boldsymbol{R}_i(\boldsymbol{Q}) - \boldsymbol{R}), \tag{13}$$

where $\mathbf{Q}$ are the normal-mode coordinates.[19] This quantity is the nuclear analogue of electron density in Density Functional Theory for electronic structure calculations.[20] We keep the roto-translational modes $\boldsymbol{Q}^{RT}$ fixed at their equilibrium position, to show the nuclear densities in the center-of-mass and principal axes frame. In Supplementary Equation 13, all the nuclear vibrational features are embodied in the squared eigenfunction factor $|\langle \boldsymbol{Q}|e_n\rangle|^2$. To estimate the vibrational wavefunctions, we expand each eigenfunction in terms of harmonic vibrational states $|\phi_{\boldsymbol{K}}\rangle$ as

$$|e_n\rangle = \sum_{\boldsymbol{K}} C_{n,\boldsymbol{K}}\, |\phi_{\boldsymbol{K}}\rangle\,, \tag{14}$$

where the coefficient $C_{n,\boldsymbol{K}}$ are estimated using the MC SCIVR method as described in the next Section, and in place of the $|\chi\rangle$ one uses the $|\phi_{\boldsymbol{K}}\rangle$.[21, 22]

Eventually, the total nuclear density for a molecule is

$$\rho_n(\boldsymbol{R}) = \sum_{i=1}^{N} \rho_{n,\boldsymbol{R}_i}(\boldsymbol{R}), \tag{15}$$

given that one-nucleus density does not overlap with the densities of the others.[19] The integral of $\rho_n(\boldsymbol{R})$ over the Cartesian coordinates equals to the number of nuclei in the molecule, and the integral of $\rho_{n,\boldsymbol{R}_i}(\boldsymbol{R})$ equals to 1. The Cartesian coordinate space representation allows the visualization of 3D nuclear densities, in a similar fashion as routinely done for electronic densities. Density distributions of other relevant observables, such as bond lengths, angles and dihedrals can be devised by straightforward modifications of Supplementary Equation 13.

To numerically evaluate Supplementary Equation 13, we set a histogram in Cartesian space, and the average value of the nuclear density in the $j-th$ bin is

$$\overline{\rho}_{n,\boldsymbol{R}_i}^{j} = \frac{1}{\Omega}\int d^{3N}\boldsymbol{Q}\, |\langle \boldsymbol{Q}|e_n\rangle|^2\, \delta(\boldsymbol{Q}^{RT}) I_{\boldsymbol{R}_i}^{j}(\boldsymbol{Q}) \quad , \tag{16}$$

where $I_{\boldsymbol{R}_i}^{j}(\boldsymbol{Q})$ is equal to 1 if, after converting $\boldsymbol{Q}$ into the corresponding Cartesian coordinate, the i-th nucleus is found within the $j-th$ bin, and zero otherwise. Ω is the volume of each bin and the density is normalized as $\Omega \sum_j \overline{\rho}_{n,\boldsymbol{R}_i}^{j} = 1$. Analogously, $\boldsymbol{R}_i$ can be substituted by bond lengths, angles and dihedrals, to calculate their quantum distributions as one-dimensional histograms and results are reported in Section IV below.

In evaluating the integral of Supplementary Equation 16 using the harmonic base expanded eigenfunctions of Supplementary Equation 14, it is convenient to employ importance-sampling Monte Carlo integration because a Gaussian sampling factor is already present in the reference state of Supplementary Equation 10. In this way, Supplementary Equation 16 becomes (dropping $\boldsymbol{R}_i$):

$$\overline{\rho}_{n}^{j} = \frac{1}{\Omega}\int \left[d^{3N}\boldsymbol{Q}\, |G(\mathbf{Q},\mathbf{\Gamma})|^2\right] \left|\sum_{\mathbf{K}} C_{n,\mathbf{K}}\bar{\phi}_{\mathbf{K}}(\mathbf{Q})\right|^2 \delta(\boldsymbol{Q}^{RT}) I^{j}(\boldsymbol{Q})\,. \tag{17}$$

The final working formula is

$$\overline{\rho}_{n}^{j} = \lim_{L\to\infty} \frac{1}{\Omega L}\sum_{l=1}^{L} \left|\sum_{\mathbf{K}} C_{n,\mathbf{K}}\bar{\phi}_{\mathbf{K}}(\mathbf{Q}_l)\right|^2 I^{j}(\boldsymbol{Q}_l) \tag{18}$$

where L independent molecular configurations $\mathbf{Q}_l$ are sampled with the Box-Muller algorithm[23] by a multivariate Gaussian distribution with null mean and variance equal to $(2\mathbf{\Gamma}/\hbar)^{-1}$. Since the harmonic contribution $\bar{\phi}_{\mathbf{K}}(\mathbf{Q}_l)$ is analytical, the computation of Supplementary Equation 18 is very fast, once the coefficients $C_{n,\mathbf{K}}$ are known. For GlyH$^+$, L is of the order of 10^8 and the error bars estimated from the square root of the variance divided by L are smaller than the plot line.

To estimate $C_{n,\mathbf{K}}$, we start from the observation that the eigenvalues of a generic Hamiltonian can be identified by the peak positions of the following Fourier transform

$$I_{\phi_{\boldsymbol{K}}}(E) = \frac{1}{\pi\hbar} Re \int_0^\tau dt \, \langle\phi_{\boldsymbol{K}}| \, e^{-\frac{i}{\hbar}\hat{H}t} \, |\phi_{\boldsymbol{K}}\rangle \, e^{\frac{i}{\hbar}Et} = \sum_n |\langle\phi_{\boldsymbol{K}}|e_n\rangle|^2 \, \delta(E_n - E) \tag{19}$$

where we choose the harmonic vibrational states $|\phi_{\boldsymbol{K}}\rangle$ as reference states. For the harmonic weights of Supplementary Equation 14 the relation $|C_{n,\boldsymbol{K}}|^2 \propto I_{\phi_{\boldsymbol{K}}}(E_n)$ holds and their sign can be determined as well.[21, 22]

We obtain the quantum time evolution and the Fourier transform in Supplementary Equation 19 by using the MC-SCIVR approach.

SUPPLEMENTARY DISCUSSION

The goal of this section is to estimate the averaged displacements in the anharmonic frame. Unfortunately there is no anharmonic equivalent to the harmonic mode displacements of Supplementary Table 3 to directly compare with. To compare the harmonic and anharmonic motion on the same ground and in a quantitative way, we calculate the standard deviation of each nucleus position for the ground and the excited O-H stretch, using the corresponding harmonic and anharmonic densities. Being the one-particle probability densities expressed in Cartesian coordinates, their standard deviations are vectors with three coordinates

$$\boldsymbol{\sigma}_{\boldsymbol{R}_i} = (\sigma_{x_i}, \sigma_{y_i}, \sigma_{z_i}) \tag{20}$$

defined according to the usual standard deviation formula

$$\boldsymbol{\sigma}^2_{\boldsymbol{R}_i} = \overline{\boldsymbol{R}_i^2} - \overline{\boldsymbol{R}_i}^2, \tag{21}$$

where the i index counts the nuclei in the molecule. Since the averages in Supplementary Equation 21 are calculated using the same sampling geometries employed for the densities calculation, these standard deviations depend on each vibrational state. As anticipated, we calculate them for the ground state and for the O-H stretch excited state density distributions. A given atom is deemed to not take part to the O-H excitation when the change of its standard deviation is negligible. Supplementary Tables 4 and 5 report in the last four columns the difference of the three standard deviation components of the excited O-H stretch state with respect to the ground state for all nuclei, both assuming a harmonic (Supplementary Table 4) or anharmonic (Supplementary Table 5) quantum approach. When the standard deviation variation is positive, nuclei are more delocalized in the excited state with respect to the ground state, while negative values are for nuclei which are more confined during the excited state motion with respect to the ground state ones. By comparing Supplementary Table 4 with Supplementary Table 5, we can see by highlighting in bold all displacements $> 10^{-6}$ Å that many more atoms are involved in the O-H stretch excitation when the anharmonic density is employed. However, the standard deviations in Supplementary Tables 4 and 5 can not be compared with the displacements in Supplementary Table 3, since the maximum elongation is not directly comparable with the standard atomic amplitude deviation.

SUPPLEMENTARY FIGURES

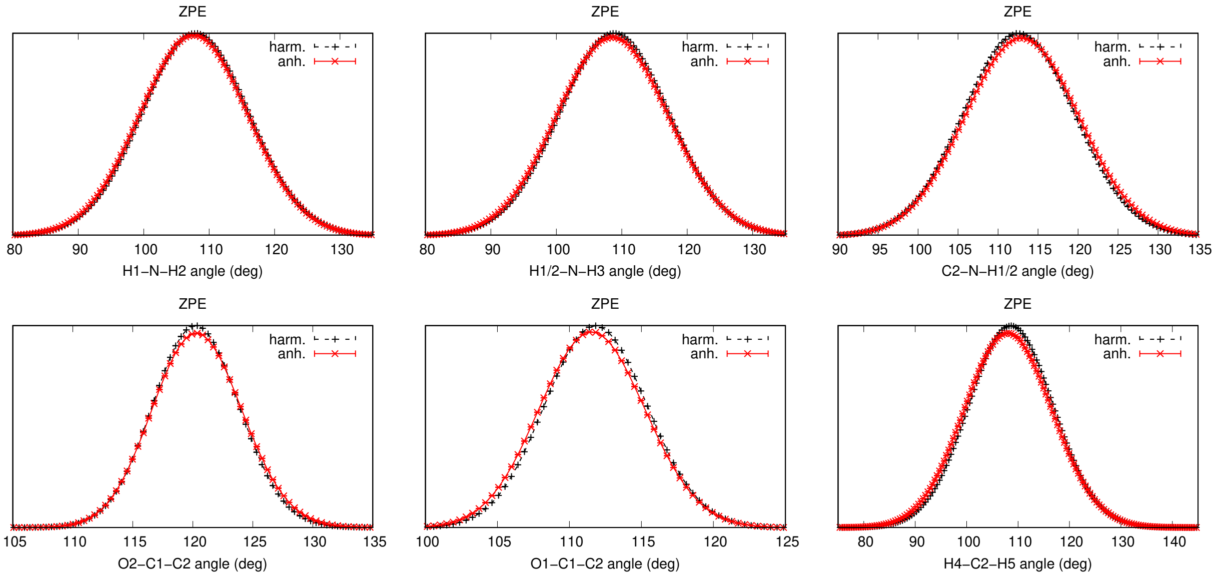

Supplementary Figure 1. Harmonic and anharmonic angle distributions for the ground vibrational state

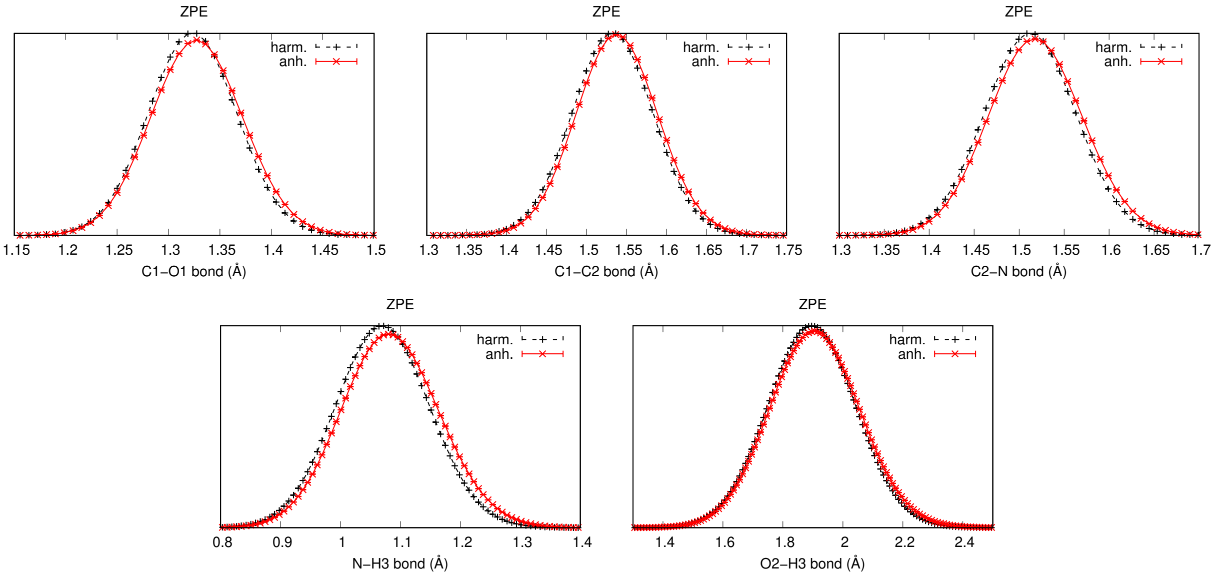

Supplementary Figure 2. Harmonic and anharmonic bond-length distributions for the ground vibrational state

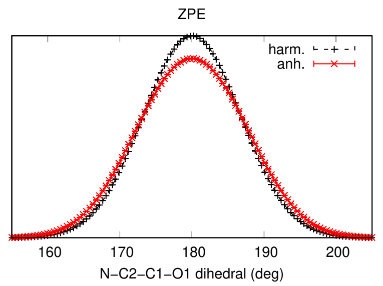

Supplementary Figure 3. Harmonic and anharmonic dihedral angle distributions for the ground vibrational state

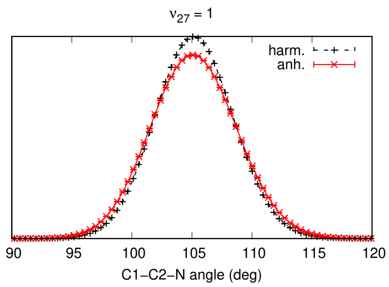

Supplementary Figure 4. Harmonic and anharmonic angle distributions for the excited O-H stretch vibrational state

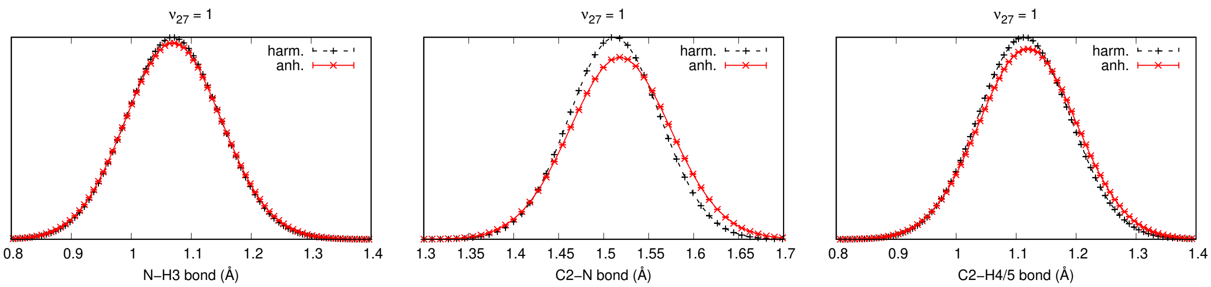

Supplementary Figure 5. Harmonic and anharmonic bond-length distributions for the excited O-H stretch vibrational state

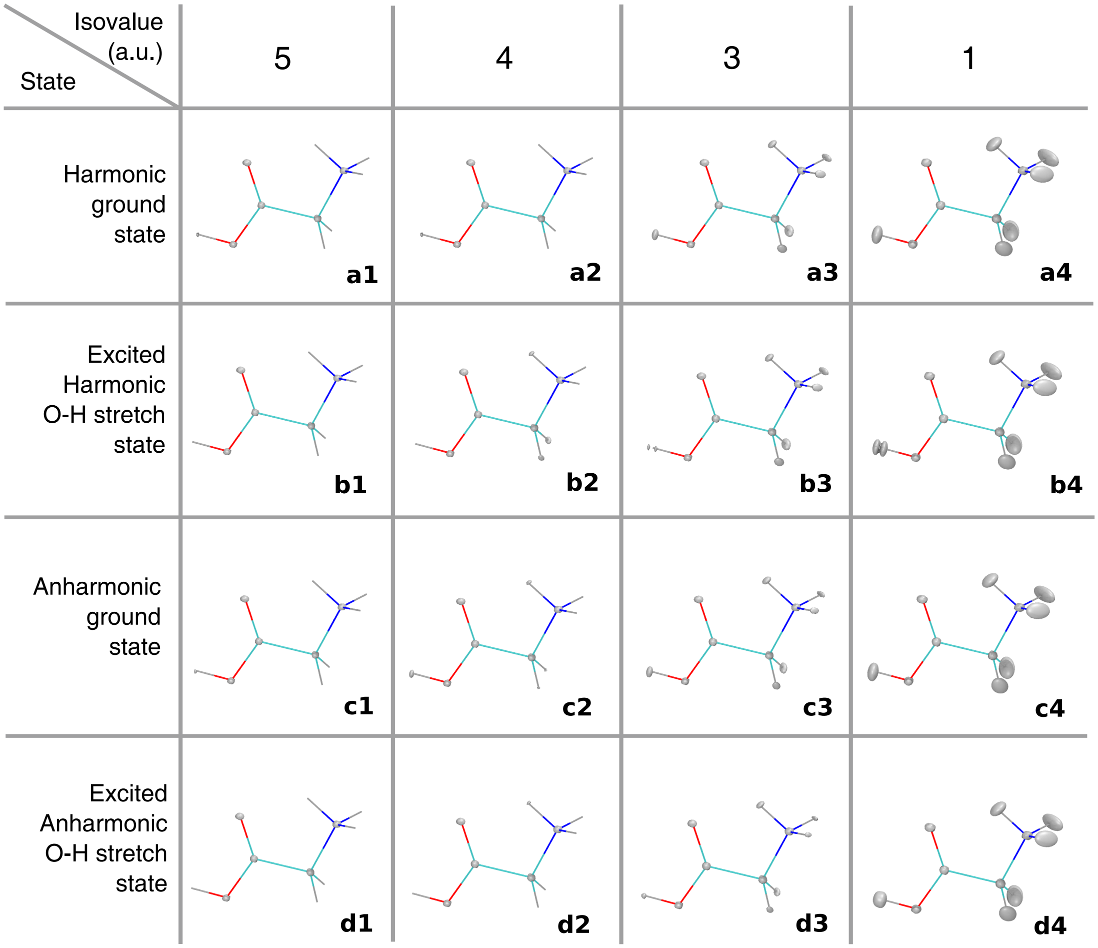

Supplementary Figure 6. **Harmonic and anharmonic nuclear density isosurfaces for the ground and the O-H stretch excited states**. For each state, listed along the first column on the left, we report four nuclear density isosurfaces plotted on the same scale. The heavier nuclei (Oxygen in red, Carbon in green, and Nitrogen in blue) show a very sharp and spatially localized density, while the Hydrogen ones have a much broader density and consequently smaller maxima. This is evident both at the harmonic and anharmonic level of calculation. The maximum values of the nuclear density for the harmonic ground state and the excited O-H stretch one are respectively 72.522 a.u. and 72.533 a.u.. The maximum values of the nuclear density for the anharmonic ground state and the excited O-H stretch one are respectively 153.457 a.u. and 142.657 a.u.. It is interesting to see that for the harmonic excited density a node is found in the O-H stretch direction (panel b4), while this is not the case for the anharmonic case (panel d4). The reason is that, in the anharmonic framework, the stretch along the O-H bond is coupled with the other normal mode degrees of freedom and it can not be reduced to a single mode one-dimensional variation along the O-H stretch direction.

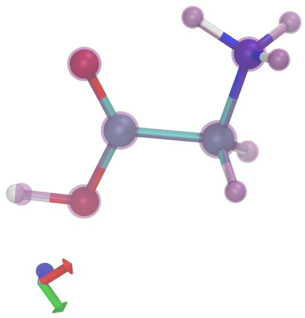

Supplementary Figure 7. Displaced geometry obtained by applying the displacements reported in Supplementary Table 3 to the equilibrium geometry. The reference frame, represented with the x red axes, the y green axes, and the blue z axes is set such that the mirror symmetry plane of the molecule lies in the x-y plane. The equilibrium geometry is represented in transparent purple and it is superimposed to the displaced geometry at the harmonic turning point which is represented with C in green, N in blue, O in red and H in gray.

SUPPLEMENTARY TABLES

Supplementary Table 1. The first nine largest (in modulus) expansion coefficients for the anharmonic ground state wavefunction. The most important contribution comes by far from the direct product of one-dimensional harmonic ground states reported in the first row, i.e. $k_i = 0$ for $i = 1, ...27$.

$\boldsymbol{k}$	k_1	k_2	k_3	k_4	k_5	k_6	k_7	k_8	k_9	k_{10}	k_{11}	k_{12}	k_{13}	k_{14}	k_{15}	k_{16}	k_{17}	k_{18}	k_{19}	k_{20}	k_{21}	k_{22}	k_{23}	k_{24}	k_{25}	k_{26}	k_{27}	$C_{n=1,\boldsymbol{k}}$
	0	0	0	0	0	0	0	0	0	0	0	0	0	0	0	0	0	0	0	0	0	0	0	0	0	0	0	9.28E-01
	0	0	0	0	0	0	0	1	0	0	0	0	0	0	0	0	0	0	0	0	0	0	0	0	0	0	0	9.82E-02
	0	0	0	0	0	0	0	0	0	0	0	0	0	0	0	0	0	0	0	0	0	1	0	0	0	0	0	-8.80E-02
	2	0	0	0	0	0	0	0	0	0	0	0	0	0	0	0	0	0	0	0	0	0	0	0	0	0	0	8.09E-02
	0	0	1	0	0	0	0	0	0	0	0	0	0	0	0	0	0	0	0	0	0	0	0	0	0	0	0	-7.68E-02
	0	0	0	0	0	0	0	0	0	0	0	0	0	0	0	0	0	2	0	0	0	0	0	0	0	0	0	6.70E-02
	0	0	0	1	0	0	0	0	0	0	0	0	0	0	0	0	0	0	0	0	0	0	0	0	0	0	0	-6.42E-02
	0	0	0	0	0	0	0	0	0	0	0	0	0	0	0	0	0	0	0	0	0	0	1	0	0	0	0	5.31E-02
	2	0	0	0	0	0	0	0	0	0	0	0	0	0	0	0	0	0	0	0	0	0	0	0	1	0	0	-4.78E-02

Supplementary Table 2. The first nine largest (in modulus) expansion coefficients for the anharmonic excited O-H stretch wavefunction. The most important contribution is given by the first row which corresponds to one quantum excitation of mode 27 (the O-H stretch one) and zero excitation for all the other modes.

$\boldsymbol{k}$	k_1	k_2	k_3	k_4	k_5	k_6	k_7	k_8	k_9	k_{10}	k_{11}	k_{12}	k_{13}	k_{14}	k_{15}	k_{16}	k_{17}	k_{18}	k_{19}	k_{20}	k_{21}	k_{22}	k_{23}	k_{24}	k_{25}	k_{26}	k_{27}	$C_{n=27,\boldsymbol{k}}$
	0	0	0	0	0	0	0	0	0	0	0	0	0	0	0	0	0	0	0	0	0	0	0	0	0	0	1	-6.09E-01
	1	0	0	0	0	0	0	0	0	0	0	0	0	0	0	0	0	0	0	0	0	0	0	0	0	1	0	3.53E-01
	0	0	0	0	0	0	0	0	0	1	0	0	0	2	0	0	0	0	0	0	0	0	0	0	0	0	0	2.48E-01
	0	0	0	0	0	0	0	2	0	0	0	0	0	0	0	0	0	0	0	0	1	0	0	0	0	0	0	-1.77E-01
	0	0	0	1	0	0	0	0	0	0	0	0	0	0	0	0	0	0	0	0	0	0	1	0	0	0	0	-1.44E-01
	0	0	0	0	0	0	0	0	0	0	0	0	0	0	0	0	0	0	0	0	0	0	0	0	0	0	2	1.29E-01
	0	0	0	0	0	0	0	1	0	0	0	0	0	2	0	0	0	0	0	0	0	0	0	0	0	0	0	-1.20E-01
	0	0	0	1	0	0	0	0	0	0	0	0	0	0	0	0	0	0	0	0	0	1	0	0	0	0	0	-1.11E-01
	0	0	1	0	0	0	0	0	0	0	0	0	0	0	0	0	0	0	0	0	0	0	0	0	1	0	0	1.02E-01

Supplementary Table 3. Cartesian atomic displacements (units are in Ångström) associated to the normal mode coordinate corresponding to the larger eigenvalue of the mass-scaled Hessian matrix evaluated at equilibrium geometry. The square root of this eigenvalue corresponds to the harmonic O-H stretch frequency equals to 3694 cm^{-1}. The magnitude of the displacements is obtained at the maximum elongation (turning point), i.e. for the potential energy equals to the harmonic ZPE value. The nuclei labeling is the same as in Fig.2a of the main text. The magnitude |**d**| of the displacement and its components along the x, y, and z Cartesian coordinates show that this normal coordinate involves mainly the O1 and H6 nuclei, and it corresponds to a stretching oscillation along their bond (for the reference frame setting see Supplementary Figure 7). The largest components are highlighted in bold.

Nuclei	x	y	z	|**d**|
N	-0.00004	-0.00002	0.00000	0.00005
C2	-0.00004	-0.00005	0.00000	0.00006
H4	-0.00007	0.00017	-0.00015	0.00024
H5	-0.00007	0.00017	0.00015	0.00024
C1	0.00019	0.00005	0.00000	0.00019
O2	-0.00006	0.00009	0.00000	0.00011
O1	**0.00521**	**0.00407**	0.00000	**0.00661**
H3	0.00025	0.00028	0.00000	0.00037
H1	0.00013	0.00001	-0.00021	0.00024
H2	0.00013	0.00002	0.00021	0.00025
H6	**-0.07225**	**-0.05759**	-0.00005	**0.09240**

Supplementary Table 4. Harmonic atomic displacement standard deviations in Cartesian coordinates (units are in Ångström). First column indicates the atom according to the labeling in the main text. Columns two, three, and four for the ground state, and five, six and seven for the O-H excited energy state density. Last four columns report the standard deviation differences between the excited and the ground ones. We highlight in bold the components $> 10^{-6}$Å in absolute value, and the corresponding nuclei.

	Harm-ZPE			Harm-27			Harm-27 – Harm-ZPE			
Nuclei	σx	σy	σz	σx	σy	σz	Δσx	Δσy	Δσz	|Δσ|
N	9.17342E-02	6.38789E-02	6.77726E-02	9.17343E-02	6.38788E-02	6.77723E-02	9E-08	-1E-07	-3E-07	3E-07
C2	7.09344E-02	7.91868E-02	6.77807E-02	7.09343E-02	7.91868E-02	6.77810E-02	-6E-08	4E-08	3E-07	3E-07
**H4**	7.10003E-02	9.01194E-02	5.25694E-02	7.10004E-02	9.01200E-02	5.25703E-02	8E-08	7E-07	9E-07	1E-06
H5	7.10029E-02	9.01121E-02	8.30739E-02	7.10030E-02	9.01116E-02	8.30744E-02	8E-08	-5E-07	5E-07	7E-07
C1	5.03531E-02	6.29595E-02	6.77754E-02	5.03533E-02	6.29596E-02	6.77753E-02	1E-07	1E-07	-1E-08	2E-07
O2	5.31665E-02	4.23752E-02	6.77742E-02	5.31665E-02	4.23751E-02	6.77744E-02	-5E-09	-3E-08	2E-07	2E-07
O1	3.07387E-02	7.42014E-02	6.77741E-02	3.07387E-02	7.42013E-02	6.77738E-02	5E-08	-2E-07	-3E-07	3E-07
H3	8.51134E-02	4.72236E-02	6.78386E-02	8.51130E-02	4.72235E-02	6.78389E-02	-4E-07	-1E-07	3E-07	5E-07
H1	1.01850E-01	6.60561E-02	5.36257E-02	1.01849E-01	6.60555E-02	5.36254E-02	-4E-07	-6E-07	-3E-07	8E-07
H2	1.01861E-01	6.60603E-02	8.19970E-02	1.01862E-01	6.60604E-02	8.19965E-02	3E-07	3E-08	-5E-07	6E-07
**H6**	1.79624E-02	6.35284E-02	6.78215E-02	1.80054E-02	6.35362E-02	6.78210E-02	**4E-05**	**8E-06**	-4E-07	**4E-05**

Supplementary Table 5. The same as Supplementary Table 4 but for the anharmonic density.

	Anharm-ZPE			Anharm-27			Anharm-27 – Anharm-ZPE			
Nuclei	σx	σy	σz	σx	σy	σz	Δσx	Δσy	Δσz	|Δσ|
**N**	9.18939E-02	6.39329E-02	6.77734E-02	9.18277E-02	6.38735E-02	6.77734E-02	**-7E-05**	**-6E-05**	1E-08	**9E-05**
**C2**	7.09466E-02	7.92046E-02	6.77830E-02	7.09383E-02	7.92270E-02	6.77823E-02	**-8E-06**	**2E-05**	-7E-07	**2E-05**
**H4**	7.09279E-02	9.02778E-02	5.25719E-02	7.10377E-02	9.02226E-02	5.25612E-02	**1E-04**	**-6E-05**	**-1E-05**	**1E-04**
**H5**	7.09305E-02	9.02702E-02	8.30876E-02	7.10400E-02	9.02150E-02	8.31010E-02	**1E-04**	**-6E-05**	**1E-05**	**1E-04**
**C1**	5.03108E-02	6.28936E-02	6.77753E-02	5.02908E-02	6.29181E-02	6.77753E-02	**-2E-05**	**2E-05**	1E-08	**3E-05**
**O2**	5.30949E-02	4.22986E-02	6.77748E-02	5.31462E-02	4.23407E-02	6.77748E-02	**5E-05**	**4E-05**	-2E-08	**7E-05**
**O1**	3.06814E-02	7.42477E-02	6.77753E-02	3.07026E-02	7.42436E-02	6.77750E-02	**2E-05**	**-4E-06**	-3E-07	**2E-05**
**H3**	8.52621E-02	4.70354E-02	6.78338E-02	8.52897E-02	4.72181E-02	6.78404E-02	**3E-05**	**2E-04**	**7E-06**	**2E-04**
**H1**	1.02020E-01	6.60615E-02	5.37098E-02	1.02056E-01	6.60629E-02	5.35979E-02	**4E-05**	**1E-06**	**-1E-04**	**1E-04**
**H2**	1.02031E-01	6.60654E-02	8.19276E-02	1.02067E-01	6.60663E-02	8.20425E-02	**4E-05**	9E-07	**1E-04**	**1E-04**
**H6**	1.78328E-02	6.37068E-02	6.78191E-02	1.76824E-02	6.33426E-02	6.78221E-02	**-2E-04**	**-4E-04**	**3E-06**	**4E-04**

SUPPLEMENTARY REFERENCES

[1] Feynman, R. P. & Hibbs, A. R. *Quantum mechanics and path integrals* (McGraw-Hill, 1965).
[2] Van Vleck, J. H. The correspondence principle in the statistical interpretation of quantum mechanics. *Proc. Natl. Acad. Sci.* **14**, 178–188 (1928).
[3] Miller, W. H. Classical S Matrix: Numerical Application to Inelastic Collisions. *J. Chem. Phys.* **53**, 3578–3587 (1970). URL `http://scitation.aip.org/content/aip/journal/jcp/53/9/10.1063/1.1674535`.
[4] Heller, E. J. Frozen Gaussians: A very simple semiclassical approximation. *J. Chem. Phys.* **75**, 2923–2931 (1981).
[5] Heller, E. J. Cellular dynamics: A new semiclassical approach to time-dependent quantum mechanics. *J. Chem. Phys.* **94**, 2723–2729 (1991).
[6] Herman, M. F. & Kluk, E. A semiclassical justification for the use of non-spreading wavepackets in dynamics calculations. *Chem. Phys.* **91**, 27–34 (1984).
[7] Tannor, D. J. *Introduction to quantum mechanics* (University Science Books, 2007).
[8] Wang, H., Manolopoulos, D. E. & Miller, W. H. Generalized Filinov transformation of the semiclassical initial value representation. *J. Chem. Phys.* **115**, 6317–6326 (2001).
[9] Zhuang, Y., Siebert, M. R., Hase, W. L., Kay, K. G. & Ceotto, M. Evaluating the accuracy of hessian approximations for direct dynamics simulations. *J. Chem. Theory Comput.* **9**, 54–64 (2013).
[10] Ceotto, M., Zhuang, Y. & Hase, W. L. Accelerated direct semiclassical molecular dynamics using a compact finite difference hessian scheme. *J. Chem. Phys.* **138**, 054116 (2013).
[11] Conte, R., Gabas, F., Botti, G., Zhuang, Y. & Ceotto, M. Semiclassical vibrational spectroscopy with hessian databases. *J. Chem. Phys.* **150**, 244118 (2019).
[12] Kay, K. G. Semiclassical propagation for multidimensional systems by an initial value method. *J. Chem. Phys.* **101**, 2250–2260 (1994).
[13] Kay, K. G. Numerical study of semiclassical initial value methods for dynamics. *J. Chem. Phys.* **100**, 4432–4445 (1994).
[14] Ceotto, M., Atahan, S., Shim, S., Tantardini, G. F. & Aspuru-Guzik, A. First-principles semiclassical initial value representation molecular dynamics. *Phys. Chem. Chem. Phys.* **11**, 3861–3867 (2009).
[15] Ceotto, M., Atahan, S., Tantardini, G. F. & Aspuru-Guzik, A. Multiple coherent states for first-principles semiclassical initial value representation molecular dynamics. *J. Chem. Phys.* **130**, 234113 (2009).
[16] Ceotto, M., Dell'Angelo, D. & Tantardini, G. F. Multiple coherent states semiclassical initial value representation spectra calculations of lateral interactions for co on cu (100). *J. Chem. Phys.* **133**, 054701 (2010).
[17] Gabas, F., Conte, R. & Ceotto, M. On-the-fly ab initio semiclassical calculation of glycine vibrational spectrum. *J. Chem. Theory Comput.* **13**, 2378–2388 (2017).
[18] Keller, J. B. Corrected bohr-sommerfeld quantum conditions for nonseparable systems. *Ann. Phys.* **4**, 180–188 (1958).
[19] Schild, A. On the probability density of the nuclei in a vibrationally excited molecule. *Front. Chem.* **7**, 424 (2019). URL `https://www.frontiersin.org/article/10.3389/fchem.2019.00424`.
[20] Parr, R. G. & Yang, W. Density-functional theory of the electronic structure of molecules. *Annu. Rev. Phys. Chem.* **46**, 701–728 (1995).

[21] Micciarelli, M., Conte, R., Suarez, J. & Ceotto, M. Anharmonic vibrational eigenfunctions and infrared spectra from semiclassical molecular dynamics. *J. Chem. Phys.* **149**, 064115 (2018).
[22] Micciarelli, M., Gabas, F., Conte, R. & Ceotto, M. An effective semiclassical approach to ir spectroscopy. *J. Chem. Phys.* **150**, 184113 (2019).
[23] Box, G. E. P. & Muller, M. E. A note on the generation of random normal deviates. *Ann. Math. Stat.* **29**, 610–611 (1958).
