# Supplementary material for: Anharmonic quantum nuclear densities from full dimensional vibrational eigenfunctions with application to protonated glycine
Source: Nat Commun. 2020 Aug 28;11:4348. doi: 10.1038/s41467-020-18211-3 (PMC7455743; doi:10.1038/s41467-020-18211-3)
Supplement: Supplementary file 4 — Description of Additional Supplementary Files [file 41467_2020_18211_MOESM4_ESM.pdf]

Description of Additional Supplementary Files

File name: Supplementary Data 1
Description : Optimized minimum geometry of protonated glycine at DFT-B3LYP/aug-cc-pVDZ level of theory used as a reference for all the calculations in the MS.

File name: Supplementary Data 2
Description: Conversion matrix for converting Cartesian coordinates to normal modes.

File name: Supplementary Code File
Description: Compressed folder containing the employed Semiclassical Nuclear Densities code and protonated glycine ground state and OH stretch excited state wavefunction data sets.
